# Deletion of low-essentiality, secretion-associated genes enhances recombinant protein production in *Komagataella phaffii*

**DOI:** 10.1186/s12934-026-03009-7

**Published:** 2026-05-11

**Authors:** Hayley Ford, Neil C. Dalvie, Timothy R. Lorgeree, Rachel M. Barry, Anaisa N. Sibel, Rachel A. Ahlmark, Harini Narayanan, Brittney C. Sunday, Charles A. Whittaker, Raghav Acharya, Carmen M. Elenberger, J. Christopher Love

**Affiliations:** 1 Department of Chemical Engineering, Massachusetts Institute of Technology, 77 Massachusetts Ave, Cambridge, MA 02139 USA; 2 Koch Institute for Integrative Cancer Research at MIT, 500 Main Street, MA 02139 Cambridge, USA

**Keywords:** Pichia pastoris, alternative host, CRISPR/Cas9, genome-wide screen

## Abstract

**Background:**

*Komagataella phaffii (K. phaffii)* is used to manufacture biologic medicines, food proteins, reagents, and materials. Despite its increasing prevalence, further improvements to its productivity would enhance its economic and operational benefits. Genomic engineering represents one approach to increase its cell-specific productivity. We hypothesized that combining the metrics for the relative essentiality of genes with biological inference for relevance to protein secretion could identify genes that, when disrupted, would improve specific productivity in the resulting strains.

**Results:**

The essentiality of genes in *K. phaffii* (NRRL Y-11430) were predicted through a genome-wide knockout screen using CRISPR-Cas9. Based on the results from this screen, we selected and subsequently disrupted the least essential genes from two gene groups heavily associated with secretion, namely those relating to the cell wall and vacuolar transport. Strains of *K. phaffii* with single gene disruptions from these gene sets showed significantly improved production of a monoclonal antibody (mAb). These strains exhibited no discernible differences in growth or apparent profiles of host cell proteins when compared to the parental strain. The best-performing strains consistently showed 2-3x enhancements in specific productivity and titers across scales (3–150 mL), culture formats (plates, flasks, bioreactors), and processing operations (batch and fed-batch).

**Conclusions:**

This study demonstrates how combining data on gene essentiality and prior knowledge of biological pathways related to a phenotypic trait of interest (here protein secretion) can inform strain engineering to enhance the trait. This study expands the catalog of genetically engineered strains of *K. phaffii* with improved productivity. These strains support the long-term goal of achieving low-cost, high-volume production of recombinant proteins using this host. Further engineering of these strains and optimization of fermentation processes could enable volumetric productivities comparable to those of other established hosts used to produce mAbs and other complex recombinant proteins.

**Supplementary Information:**

The online version contains supplementary material available at 10.1186/s12934-026-03009-7.

## Background

There is a growing global demand for high-quality recombinant proteins, including biopharmaceuticals, reagents, food ingredients, and industrial enzymes [1, 2]. Industrial-scale, cost-effective production of these proteins is necessary to sustain growing supply chains and increase global accessibility. Eukaryotic microorganisms provide several advantages for improving the space-time yield for production and recovery of recombinant proteins. These include their rapid growth rates, their ability to secrete proteins into a culture medium (simplifying recovery of products), their relatively small profile of secreted host proteins, and their substantial robustness in bioprocesses [3–8]. *Komagataella phaffii (K. phaffii)* (commercially known by the tradename “Pichia” or “Pichia pastoris” [9]) is one such host and is commonly employed to produce proteins used in food, materials, subunit vaccines, and biopharmaceuticals, including FDA-approved therapeutics [10]. For some proteins, such as monoclonal antibodies (mAbs), demonstrated space-time yields in *K. phaffii* are not yet comparable to yields from cell lines like Chinese Hamster Ovary (CHO) that have been optimized over the last 30 years through cellular and process engineering [5, 6, 11, 12]. Achieving comparable volumetric productivity with alternative hosts, such as *K. phaffii*, could enable additional economic and operational benefits for agile and flexible manufacturing [3, 13].

One approach to enhance the specific productivity of cells is through genetic engineering. Modifying the genome of manufacturing hosts to improve productivity is well-established for both traditional and alternative manufacturing hosts [7]. Thus far, the genetic engineering of non-model organisms (including *K. phaffii*) has been explored primarily using two approaches. The first approach is to induce random mutations [14]. This approach often results in large numbers of strains that lack beneficial characteristics and it can be difficult and costly to characterize the best-performing ones [15]. The second approach uses specific hypotheses to guide small numbers of rational-based genetic edits (like gene disruption, addition, or modification) [16–19]. Hypothesis-driven genetic editing requires detailed knowledge of the host strain’s genome that is often limited for non-model organisms [15]. Additionally, both engineering strategies can be time-consuming and resource intensive. Alternatively, screening using modern methods for genome editing (e.g., CRISPR-Cas9) can enable streamlined genome-scale screens to evaluate the influence of genes on cellular functions using limited prior knowledge of the host organism [20, 21].

In *K. phaffii*, targeted disruption of native genes is one strategy for genetic engineering that can enhance production of recombinant proteins [18, 22–25]. Here we report an approach to select genetic perturbations for improved production of recombinant proteins in *K. phaffii*. We used CRISPR-Cas9 to screen and evaluate the essentiality of genes in *K. phaffii* and then used those data, in conjunction with prior knowledge of pathways associated with secretory function, to select gene candidates for disruption. The selected candidates were evaluated using a CRISPR screen comprising arrayed guide RNAs (gRNAs). We show that disrupting genes identified using this approach significantly improved the productivity of strains producing trastuzumab, a model therapeutic mAb, across different formats for cultivation (plates, flasks, and reactors).

## Materials and methods

### Gene essentiality and gene groups

The genome-wide essentiality screen was performed as reported previously [26] with two modifications: (1) all genes in the genome were screened, rather than a subset of secretome genes, and (2) essentiality was indirectly estimated using the log-fold change in abundance between the untransformed library of guides and the Cas9 + library.

Broadly summarizing the protocol: for every coding sequence in the genome of *K. phaffii*, we designed five single guide RNAs (gRNAs) that targeted sequences within the first 500 bp of the coding region. This library of guides was propagated in *Escherichia coli* (*E. coli*) and then transformed into a strain of *K. phaffii* with a genomically integrated Cas9 enzyme (Cas9 + strain). After transformation and cell growth, cells were harvested into a pooled cell bank and nested PCR was used to amplify the gRNA cassettes, add barcodes, and attach sequencing handles. The resulting amplicon library was sequenced using an Illumina HighSeq 2000 with a custom primer for the gRNA sequence and a TruSeq i7 index primer. The number of reads for each gRNA within the amplicon library was compared to the number of reads from the original library of untransformed guides and the fold-change difference for each guide was calculated. A one-sided Kolmogorov-Smirnov (KS) test was performed on the log fold change for each gene. Essentiality estimates are reported as the -log_10_ of the KS test p-value [26].

Gene groups were identified using a previously published genome annotation [27]. Unannotated genes were grouped based on their homology with annotated genes from the *Saccharomyces* gene database [28].

### Strains and vectors

The screen for essentiality used a wild type strain of *K. phaffii* (NRRL Y-11430) with Cas9 integrated at GQ67_01884 under the control of the P_ENO1_ promoter as described previously [26].

The base strain referenced (and the strain from which all engineered strains were derived) was a modified variant [RCR2_D196E, RVB1_K8E; AltHost Research Consortium Strain S-63] of wild-type *K. phaffii* (NRRL Y-11430) described previously [29].

Recombinant trastuzumab was codon optimized for *K. phaffii* using the Thermo Fisher Scientific codon optimizer. The heavy chain and the light chain were integrated into a single custom vector oriented convergently [AltHost Research Consortium A Vector, D-1073]. Trastuzumab heavy chain was cloned with the methanol inducible promoter, P_AOX1,_ while trastuzumab light chain was cloned with the methanol inducible promoter P_DAS2_. Both heavy and light chain were expressed with the pre-OST1/pro-alpha signal peptide adapted from *Saccharomyces cerevisiae* (*S. cerevisiae).* Four amino acids (EAEA) were included between the signal peptide and the protein sequence to improve cleavage of the signal peptide [30].

The native codon sequence was used for the recombinant human serum albumin (HSA). This sequence was also cloned into a custom vector used previously [AltHost Research Consortium L Vector, D-17] [26]. The HSA plasmid used the P_AOX1_ promoter and was expressed with the α-mating factor signal peptide from *S. cerevisiae*. 

Both the trastuzumab and HSA plasmids were linearized within the P_AOX1_ promoter for integration at the AOX1 locus. *K. phaffii* was transformed with linear DNA by electroporation as described previously [31]. 5 µg of linear DNA was used for each transformation and plated cells were recovered with the appropriate antibiotics (Zeocin™ or Kanamycin or both). The DNA itself was integrated into the genome through DNA-mediated multicopy tandem integration that made use of homology arms for repeated integration at the linearized promoter.

In strains with targeted genetic modifications, genes were disrupted using a custom CRISPR-Cas9 vector [AltHost Research Consortium R Vector] [31] and knockout fragment [32] as described previously. Two CRISPR guides were selected for each intended knockout. Guides were designed to be as close as possible to the beginning of the gene coding region. In the event one guide failed, we used the second guide. When Cas9-mediated double-stranded DNA cleavage was detected but no viable knockout strains were recovered (i.e., only amino acid deletions at the CRISPR guide site), that gene was reclassified as essential regardless of the essentiality estimates from the genome-wide screen. Integration of the knockout fragment was confirmed for each strain by Sanger sequencing of PCR-generated fragments (GENEWIZ from Azenta, Quintara Biosciences).

These targeted gene disruptions were performed in strain backgrounds with stably integrated recombinant protein expression vectors that showed high expression of recombinant proteins in the base strain (S-63) to reduce confounding effects on protein production caused by varied copy number.

Plasmid sequences for the recombinant proteins and the CRISPR-Cas9 enzyme are included in the supplemental information (CRISPR-Cas9 enzyme plasmid file includes the guide for JSN1disruption [AltHost Research Consortium R Vector, D-1266]). Supplemental tables are also included with the lists of guide RNAs used (Tables S7 and S11).

### Selecting protein-containing strains with high expression levels

Base strains with integrated recombinant proteins were scouted for optimal copy number prior to genetic engineering. Eight clones were selected after the transformation of recombinant protein into the base strain. These clones were then cultivated using the 3 mL screening culture method described below. Harvested supernatant samples were analyzed by SDS-PAGE and the clone with the highest level of protein expression was selected as the parent strain for further engineering.

### Strain cultivation: 3 mL screening cultures

Small-scale screening cultures (3 mL) were performed in 24-well deep well plates (10 mL V-bottom from Axygen^®^) at room temperature (~ 23 °C) on plate shakers (3 mm orbit diameter) at 600 rpm. Cultivations were started from streaked YPD plates and grown overnight (~ 16 h) in 5 mL YPD. Plates were inoculated at an optical density (OD_600_) of 0.1 in complex media (1.0 M potassium phosphate buffer, pH 6.5, 1.34% yeast nitrogen base without amino acids, 1% yeast extract, and 2% peptone).

Screening cultures for protein expression from strains with single edits were grown for 24 h with 4% v/v glycerol as a carbon source. After 24 h, the cultures were pelleted and resuspended in fresh complex media containing 1.5% v/v methanol and 10 mM glutathione. Glutathione is a common media additive for CHO media when producing antibodies [33]. Cells were cultivated in the fresh media for 24 h, after which the supernatant was harvested by centrifugation.

Extended 3 mL cultures for strains with dual gene disruptions were also grown for 24 h in media with 4% v/v glycerol as a carbon source to start. After 24 h of outgrowth, the cells were pelleted and resuspended in fresh media containing 1.5% v/v methanol and 10 mM glutathione. Cultivation in the fresh media lasted for 48 h with a spike of 1.5% v/v methanol 24 h into the production period. The supernatant was again harvested by centrifugation.

Strains were cultivated in triplicate for all plate cultivations and OD_600_ was measured every 24 h. Mid-cultivation samples and the supernatant collected at harvest were analyzed using assays to quantify and characterize proteins as described below.

### Strain cultivation: 50 mL flask cultures

50 mL cultures were performed in 250 mL baffled shake flasks (Kimble^®^, PYREX^®^). Flasks were cultivated in an incubator at 25 °C, 300 rpm, with an orbit diameter of 19 mm. Cultivations were started from streaked YPD plates and grown overnight (~ 16 h) in 5 mL YPD. Flasks were inoculated at an optical density (OD_600_) of 0.1 in complex media (1.0 M potassium phosphate buffer, pH 6.5, 1.34% yeast nitrogen base without amino acids, 1% yeast extract, and 2% peptone).

As was done for the 3 mL cultures, cells were initially grown for 24 h in media containing 4% v/v glycerol as a carbon source. After this outgrowth period, the cultures were pelleted via centrifugation, and the pellets were resuspended in fresh media containing 1.5% v/v methanol and 10 mM glutathione. The 50 mL extended cultures were cultivated in the fresh media for a total of 72 h before harvest via centrifugation. Every 24 h during production, 750 µL of the culture was removed for analysis and 1.5% v/v methanol was added to the remaining culture.

Strains were cultivated in triplicate. The 750 µL samples collected every 24 h were used to measure the OD_600_. The remainder of each sample and the supernatant collected at harvest were analyzed using assays to quantify and characterize proteins as described below.

### Strain cultivation: fed batch bioreactor

The reactor campaign was conducted using the Sartorius AMBR^®^250 system. A modified fed-batch protocol was used. Cells were inoculated at 0.4 OD_600_ in 75 mL of defined media (STx-006, Sunflower Therapeutics, PBC) with 1% glycerol. The outgrowth stage with an exponential glycerol feed lasted 35.5 h and delivered a total of 16.6 mL of glycerol. At the end of this outgrowth stage, we added an additional 75 mL of defined media (STx-006) for a total working volume of approximately 150 mL. The process concluded with a methanol-fed production stage lasting five days wherein a bolus dose of 1.5% v/v methanol was added every six hours with the first dose administered at 35.5 h. 28 h into the outgrowth stage, the stir speed for the reactors was set manually to 2000 rpm, and dissolved oxygen (DO) was passively monitored. Throughout the campaign pH was held at 6.5, and temperature was maintained at 25 °C.

### Assays for protein quantification and characterization of recombinant proteins

*SDS-PAGE.* SDS-PAGE was carried out using Novex 12% Tris-Glycine Midi Gels (Thermo Fisher Scientific) according to the manufacturer’s recommended protocol. Gels were stained with Instant Blue Protein Stain (Thermo Fisher Scientific) or ReadyBlue^®^ Protein Gel Stain (Sigma-Aldrich). 

*Titer.* Titers of trastuzumab from all 3 mL plates and flask cultivations for strains with vacuolar edits were measured by Protein A biolayer interferometry (Sartorius Octet^®^) as described previously [34]. All titers of HSA, titers of trastuzumab from the flask cultivations with the cell wall-derived edits, and titers of trastuzumab from the reactor run were measured using Agilent Infinity 1260 High Performance Liquid Chromatography as described previously [35]. HSA was quantified using a reverse-phase column (Agilent Technologies, Cat# PL1912-3501), while trastuzumab was quantified using a Biomonolith protein A column (Agilent Technologies, Cat#5190-6903).

### Analytical methods

Specific productivity was calculated as supernatant titer (mg/L) divided by cell density (OD_600_) at a given timepoint.

Cell density (OD_600_) was measured using the Tecan Infinite M Nano+ plate reader for plate cultures, and the Thermo Scientific™ GENESYS™ 150 for flask cultures.

Fold changes were calculated as the ratio of the mean result for the strain of interest to that of the strain to which it was compared.

### Statistical analysis

Strain titers and specific productivities were analyzed using a one-way ANOVA analysis on the averages across replicates. Growth profiles and OD measurements were analyzed using a two-way ANOVA analysis on the averages for each strain at a given time point. Essentiality profiles were analyzed using the Kolmogorov–Smirnov test and the Mann–Whitney test. Reported correlations between variables were analyzed using the Spearman Rank Correlation test.

## Results

### Essentiality characterized by genome-wide knockout screen

Each gene exhibits a distinct degree of essentiality for cell survival under laboratory-controlled conditions. We hypothesized that quantitative estimates of essentiality could serve as a predictive metric for cellular tolerance of gene edits, and, when used in conjunction with known gene functions, could guide the rational selection of targets for genetic editing to improve production of recombinant proteins.

**Fig. 1:**
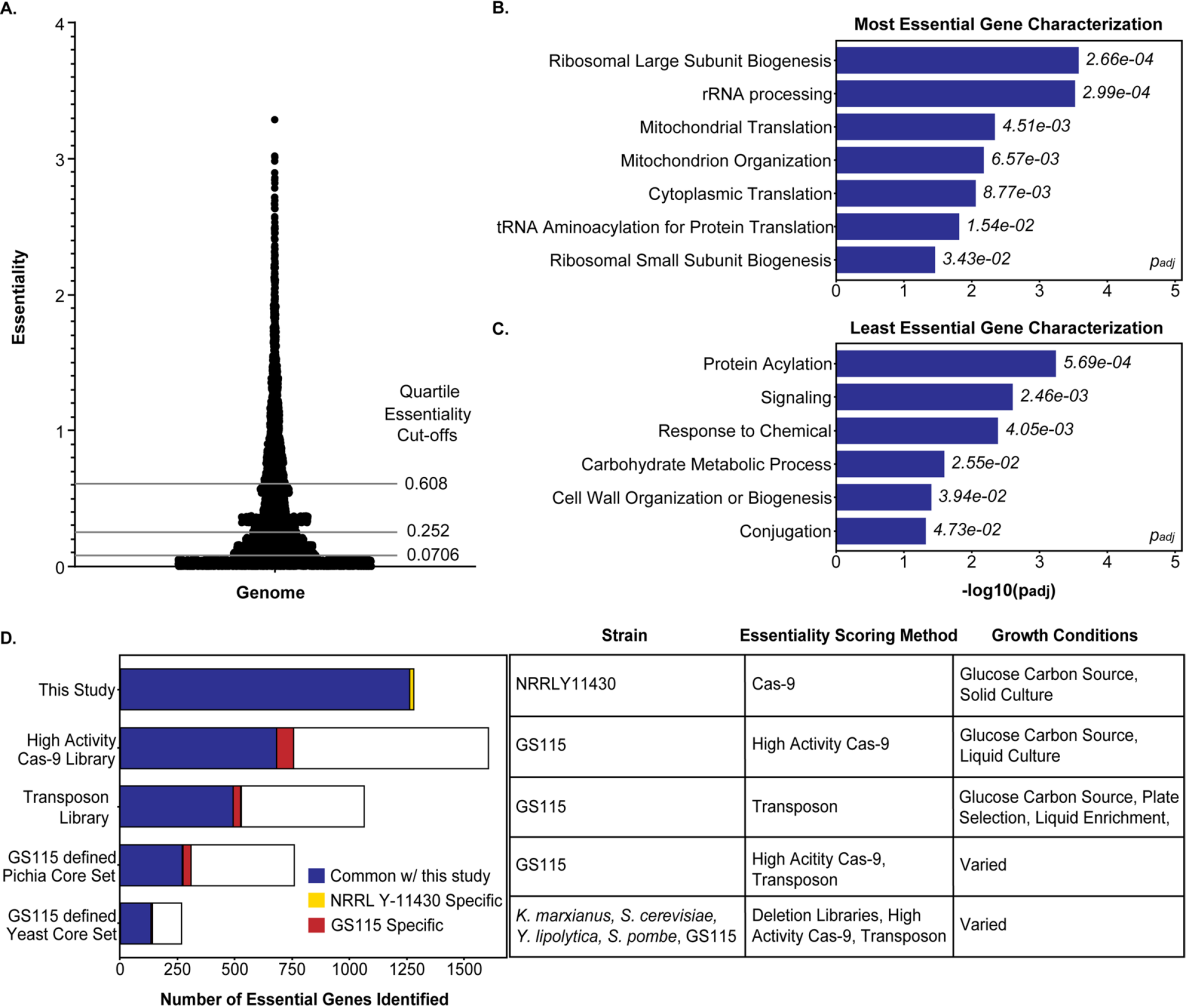
Characterization of whole genome knockout screen in *K. phaffii*. **A** Essentiality of all genes in the genome of *K. phaffii* (NRRL Y-11430) with essentiality cut-offs at each quartile (1280 genes per quartile). **B** Most represented gene groups in the 1,280 most essential genes (top quartile) as defined by Over-Representation Analysis (ORA). **C** Most represented gene groups in the 1280 least essential genes (bottom quartile) as defined by ORA. **D** Commonality comparison of reported essentiality screens in *K. phaffii* (NRRL Y-11430 and GS115) and related organisms (*K. marxianus*,* S. cerevisiae*,* Y. Lipolytica*,* S. pombe*). Bar lengths represent the number of essential genes identified by each screen. Colors denote the number of organism-specific genes identified by each screen and the number of genes in common between the reported screen and this study. References: High Activity Cas9 Library [21], Transposon Library [36], GS115 defined Pichia Core Set [21], GS115 defined Yeast Core Set [21]

To address this hypothesis, we first estimated the essentiality of the genes in *K. phaffii* (NRRL Y-11430). The essentiality of genes in *K. phaffii* has been assessed in literature for the related GS115 strain cultivated in glucose using a high-activity CRISPR-Cas9 screen in liquid culture [21] and a transposon-based approach that used plate selection and liquid enrichment [36]. We previously evaluated the essentiality of genes found in the secretome of the NRRL Y-11,430 strain using a CRISPR-Cas9 screen on cells grown with glucose on solid media [26]. We expanded this approach here to perform a genome-wide screen for gene essentiality (Fig. 1). A strain with a genomically integrated Cas9 enzyme was transformed with library of plasmids that encoded five gRNAs for every gene. The resulting clones from this transformation were sequenced and gene essentiality was then indirectly estimated based on gRNA abundance. Strains from the transformed library that retained a high abundance of a gRNA plasmid were scored with a lower estimated value of essentiality. That is, transformed strains with high abundance of the gRNA after screening indicate genes that upon disruption have limited impact on the fitness or survival of the strain. The use of multiple guides per gene was intended to reduce misclassification of essentiality caused by the variable efficiency of individual gRNAs [37]. We assigned a quantitative essentiality estimate to each gene by calculating the log-fold differences in gRNA between the transformed and untransformed library (Table S1). We then classified the genes into quartiles (Fig. 1A). Similar screens for essentiality across multiple organisms have suggested ~ 20–30% of yeast genes are essential [21]. We used an Over-Representation Analysis (ORA) [38] of the quartiles with respect to defined gene groups [27] to identify pathways that were statistically over-represented in each quartile (Fig. 1B, C). We further characterized the quartiles by evaluating the percentage representation of all gene groups within each quartile (Tables S2 S3).

The most essential quartile showed significant over-representation in gene groups associated with ribosomal processes (including subunit biogenesis and rRNA processing) and translation processes (Fig. 1B). These same classes of genes were also highly essential in other published screens for essentiality in *K. phaffii* [21, 36] as well as other organisms [39, 40]. In addition to having statistically over-represented gene groups for translation processes, the most essential gene group contained roughly three times more genes associated with transcription and translation (including DNA and RNA processes) than the least essential gene group (~ 9.2% compared to ~ 3.1%) (Table S2, S3).

The least essential quartile had elevated numbers of genes from pathways we would expect to be less essential under the specified growth conditions. These pathways included certain genes associated with post-translational modifications, signaling, cellular responses, metabolic processes, and cell wall formation (Fig. 1C). Broadly, the bottom quartile contained about 1.5x the number of genes associated with cellular response pathways (13.4% vs. 9.3%) and about 3x the number of genes in signaling pathways (3.4% vs. 1.2%) when compared with the top quartile (Table S2, S3). Finally, the most essential gene group showed a lower percentage of unclassified genes (44.4%) (Table S2) than the least essential gene group (57.1%) (Table S3). This result is consistent with the expectation that essential genes are more likely to be annotated or have a known function due to their higher conservation across species [41] and their involvement in essential pathways, which tend to be better characterized functionally [42].

We then compared our results for the NRRL Y-11,430 strain of *K. phaffii* with reports on genome-wide essentiality for GS115 (Fig. 1D) [21, 36]. There was ~ 50% homology between our study and each of the other studies in *K. phaffii* (see Tables S4, S5 for lists of genes conserved between sets). Conservation of ~ 50% of genes was also consistent with the commonality observed between each of the other comparable studies. The observed variations may result from differences in strain backgrounds, experimental methods, and computational analyses.

### Disruption of cell wall genes with low essentiality improved production of recombinant protein

We hypothesized that genes with low predicted essentiality would represent promising targets for genetic disruption since such edits should have minimal effects on the fitness of the cells. We further hypothesized that disrupting genes in cellular pathways directly associated with secretion could improve cell-specific productivity. Prior studies have shown that large, complex proteins, such as mAbs, can accumulate in the endoplasmic reticulum (ER) and the secretory pathway specifically [7, 43], and that modifications of individual secretion-associated genes can enhance recombinant protein production [14, 18, 22–25, 44–49]. We elected to specifically target genes related to the cell wall and vacuolar transport since these systems present potential barriers for transport of proteins through the secretory pathway [43].

Fungal cell walls contribute significantly to maintaining structural durability, enabling high-density growth in fermenters. Despite the critical role of the cell wall for robustness in fermentation, we hypothesized that the relatively limited porosity of the cell wall may hinder the secretion of large and complex proteins [50]. Supporting this idea, microscopy of cells expressing a fluorescently stained, catalytically inactive botulinum neurotoxin (BoNT) (~ 160 kDa) showed protein aggregation at the cell wall (Image S1, Video S1). We postulated that disrupting select cell wall components could increase cellular permeability, specifically for large recombinant proteins, without compromising the integrity of the cell wall. This hypothesis is consistent with prior studies that have reported increased production/secretion of recombinant proteins in *S. cerevisiae* [46–49] and *K. phaffii* [14, 18, 22, 26] that have modified cell walls or cell wall proteins.

**Fig. 2:**
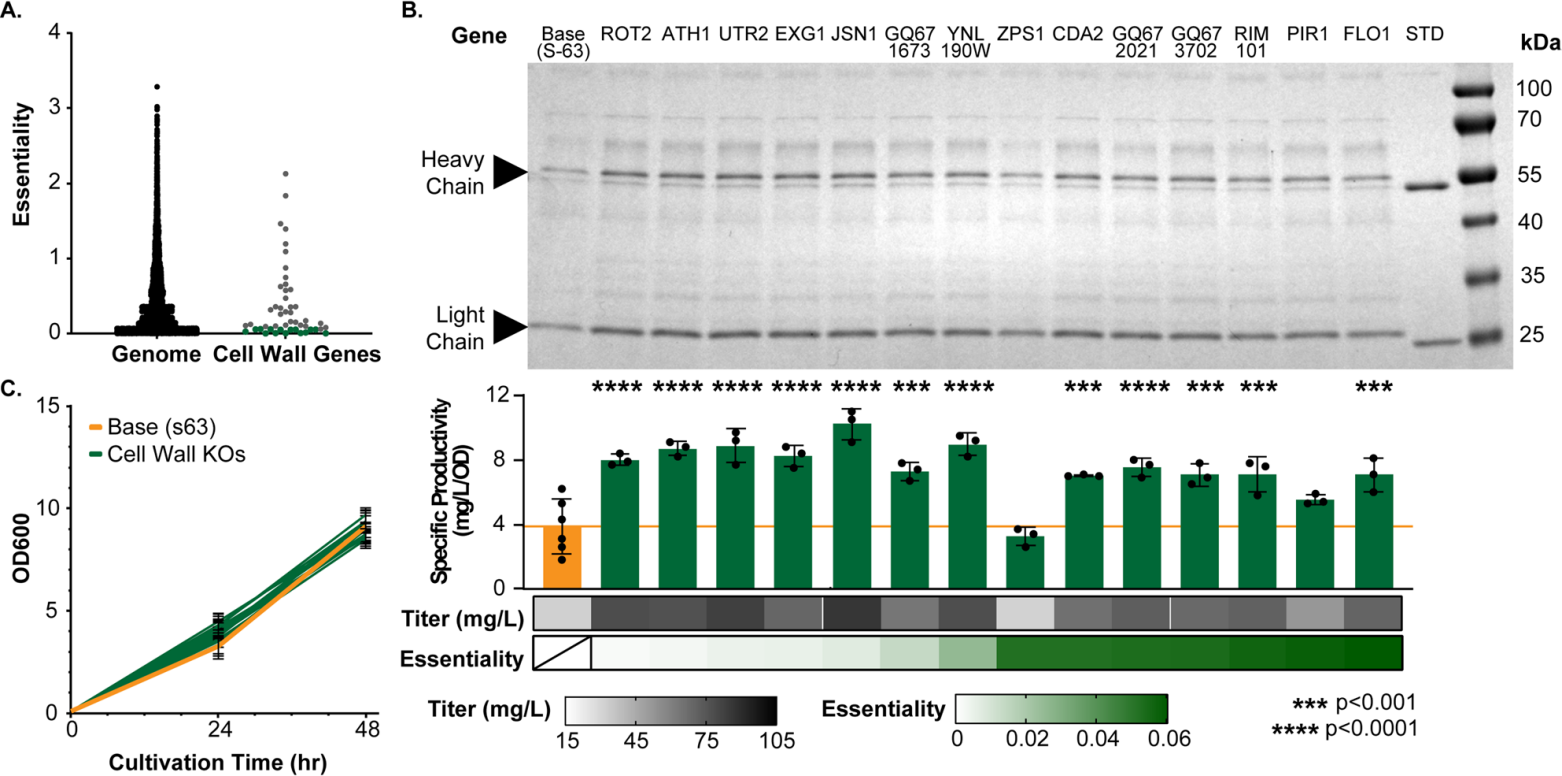
Characterization of the least essential cell wall gene disruptions. **A** Essentiality of genes in the identified cell wall group. **B** Effects of targeted cell wall gene disruptions on the secretion of glycosylated IgG1 (trastuzumab). Results include SDS-PAGE gel of supernatants from 3 mL cultures, corresponding specific productivities (mg/L/OD600), titers (mg/L), and gene essentiality scores. Error bars represent standard deviation. **C** Optical densities (OD600) of 3 mL cultures as a function of time. Error bars represent standard deviation. Statistical significance was determined using an ordinary one-way ANOVA test followed by Dunnett’s multiple comparison test to compare each strain to the base strain.

We compiled the genes in the *K. phaffii* genome that are known components of the cell wall or are involved in its construction or maintenance (Table S6). These genes included mannoproteins, GPI anchor proteins, proteins involved in α- and β-glucan assembly, and transcription and translation factors that regulate construction of the cell wall. This list was ranked by essentiality and, interestingly, showed a relatively low essentiality profile when compared to the complete genome (Fig. 2A), though the difference was not statistically significant (Table S8). Further supporting the observation that the class of cell wall genes is broadly less essential, our ORA analysis showed significant over-representation of genes related to organization and biogenesis of the cell wall in the least essential quartile (Fig. 1C).

The 15 least essential cell wall-related genes were selected for disruption (Table S7). We successfully disrupted 14 of the 15 selected genes in the base strain (S-63). One gene, MCD4, was not recovered as a viable knockout and was subsequently reclassified as essential. This example suggests that multiple factors, including gene accessibility, repair mechanisms, and environmental conditions used, could cause misclassification of essential genes. Nonetheless, the general success of targeting genes for disruption here (Figure S1), along with the concordance with other reported screens of essentiality in this organism (Fig. 1), indicate the overall effectiveness of the screen applied here to assess essentiality on a genome-wide scale. We then attempted to knockout these 14 genes in two strains producing recombinant proteins: one producing bicistronically integrated trastuzumab and one producing human serum albumin (HSA). Both proteins are model therapeutics; mAbs are heterodimeric glycoproteins that require correct folding, disulfide bond formation, and multimeric assembly [51] whereas recombinant HSA is single-chain protein that can be produced in multiple expression hosts [52]. All 14 genes were successfully knocked out in the trastuzumab producing strains and 13 of the 14 were successfully knocked out in the HSA producing strains.

The resulting engineered strains were cultivated at plate scale for 48 h. For the trastuzumab-producing strains, the harvested supernatant showed comparable profiles of host cell proteins and growth across all strains (Fig. 2B, C). Titers and specific productivities differed significantly from the base strain for all mAb-producing strains with cell wall knockouts, except ΔZPS1 and ΔPIR1 (Fig. 2B). Within the subset of low-essentiality genes, no correlation was observed between essentiality and specific productivity. ΔROT2, ΔATH1, ΔUTR2, ΔEXG1, and ΔYNL190W all showed a greater than 2-fold increase in cell-specific productivity and titer. The highest-producing knockout strain was the ΔJSN1 strain. This strain showed a 2.6-fold increase in cell-specific productivity and a 2.5-fold increase in recombinant protein titer.

JSN1 is annotated as a member of the Pumilio and FBF (PUF) protein family, characterized by the presence of Pumilio homology domains [27]. In *S. cerevisiae*, JSN1 (also known as PUF1) “interacts with mRNAs encoding membrane-associated proteins” and is involved in Arp2/3 complex localization (the Arp2/3 complex playing a critical role in actin cytoskeleton regulation) [28]. We hypothesize that productivity improvements shown by this knockout strain may result from a reduction in membrane-associated proteins, increasing membrane permeability. Alternatively, the disruption of JSN1 may alter the cell’s actin cytoskeleton, which, in turn, may increase cell wall permeability or modify protein trafficking. As a member of the PUF protein family, JSN1 may also enhance the mRNA stability of the recombinant protein itself, thereby facilitating improved translation [53].

ROT2, UTR2, EXG1, and YNL190W are all involved in the synthesis of the cell wall, with UTR2, EXG1, and YNL190W specifically contributing to beta-glucan assembly [27]. We hypothesize that disrupting these genes may alter the structure of the glucan layer surrounding the cell. This change could directly increase cell wall porosity or reduce physical obstructions along the secretory pathway. It could also impair the cell’s ability to retain mannoproteins, which may play a direct role in determining cell wall permeability [54]. Alternatively, all four of these genes are found in the secretome of *K. phaffii* [26] and may be in direct competition with heterologous proteins for translational and secretory resources. Disrupting these genes may reduce the secretory burden of the cell, increasing its capacity for production of recombinant proteins.

In *S. cerevisiae*, ATH1 is a trehalase that localizes to the cell wall [28]. We hypothesize that disrupting ATH1 may enhance secretion of recombinant proteins by reducing the number of cell-wall localized proteins or by decreasing transportation burden to the cell surface. The ATH1 knockout may, however, have metabolic benefits as well. Loss of ATH1 activity may lead to increased levels of intracellular trehalose. Trehalose has been shown to stabilize proteins when the cell is under stress [55], promote the clearance of misfolded proteins, and prevent aggregation [56]. Because cells producing large quantities of recombinant proteins often experience elevated levels of cellular stress and misfolded proteins [57], an increased level of trehalose may positively impact the ability of the cell to function when producing large quantities of recombinant protein. Trehalose as an excipient has also been shown to stabilize trastuzumab specifically [58]. These increased stabilizing effects may be occurring intracellularly as previously hypothesized, or the lack of the trehalase may lead to increased levels of extracellular trehalose throughout the cultivation, which could act as a stabilizer for the secreted proteins. Given ATH1’s metabolic role, this strain’s viability and performance in minimal or defined media may deviate more from our observations than the other strains presented in this study.

None of the HSA-producing strains with cell wall disruptions showed any beneficial modulation of titer or cell-specific productivity (Figure S2). The observed results suggest that the rate-limiting steps for the production of HSA differ from those for producing trastuzumab. Similar to the trastuzumab-producing strains, these strains also showed no discernible differences in growth or profiles of secreted host cell proteins, indicating these gene edits were well-tolerated across varied genetic backgrounds.

### Disruption of vacuolar genes with low essentiality improved production of recombinant protein

Reducing the flow of proteins through the vacuolar pathway can reduce degradation of desired products and increase protein secretion [24], indicating that the vacuolar pathway can act in direct competition with the secretory pathway. It has been demonstrated that knocking out genes in the vacuolar pathway can improve productivity in *K. phaffii* [23–25, 44, 45]. One available commercial strain of *K. phaffii* includes two vacuolar knockouts (PichaPink™) [25]. We hypothesized that this group of genes may contain additional proteins that, when disrupted, would improve production of recombinant proteins with minimal impact on host physiology.

**Fig. 3:**
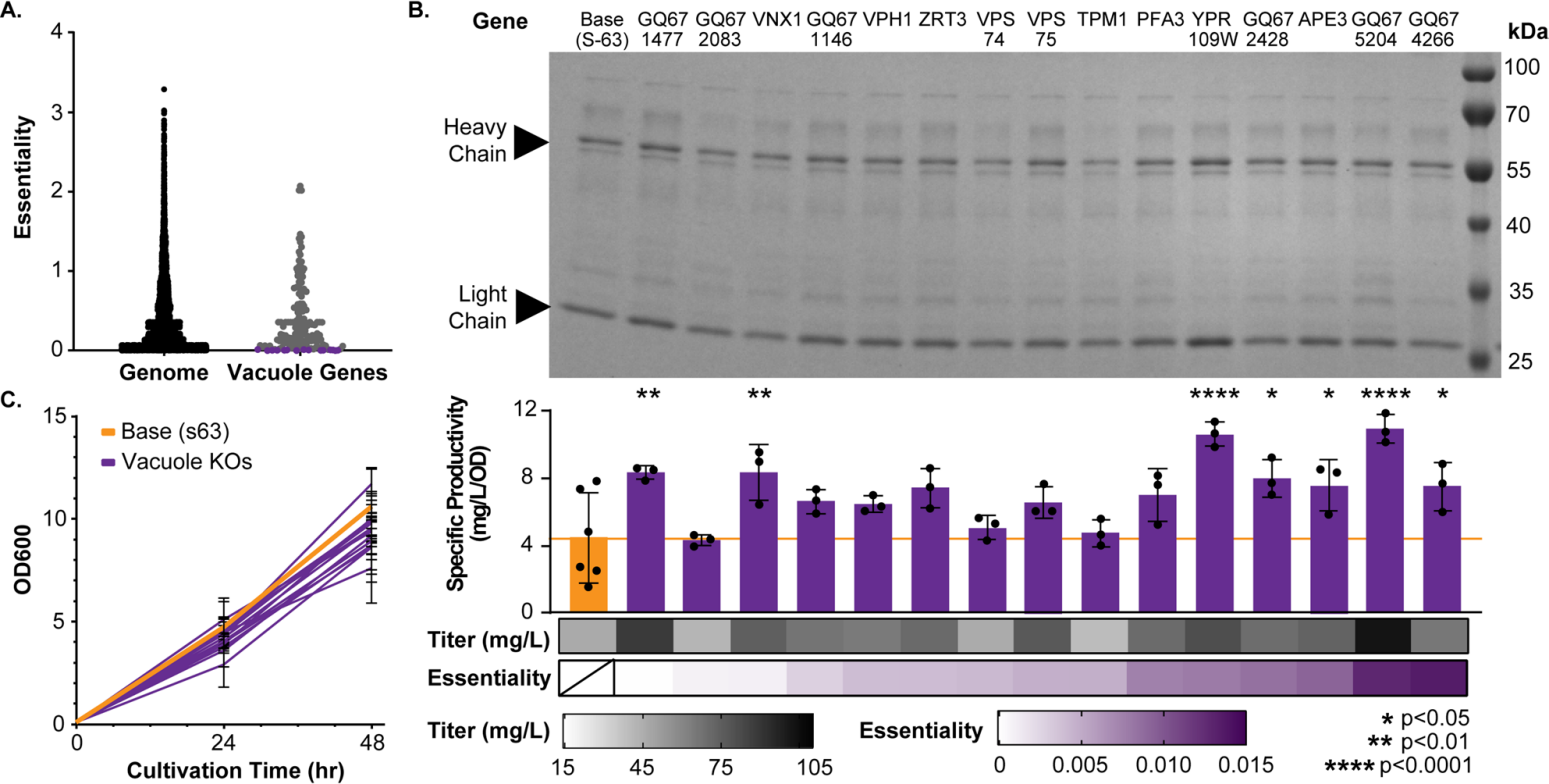
Characterization of the least essential vacuolar gene disruptions. **A** Essentiality of genes associated with the vacuolar pathway. **B** Effects of targeted vacuolar gene disruptions on the secretion of glycosylated IgG1 (trastuzumab). Results include SDS PAGE gel of supernatants from 3 mL cultures, corresponding specific productivities (mg/L/OD600), titers (mg/L), and gene knockout essentiality scores. Error bars represent standard deviation. **C** Optical densities (OD600) of 3 mL cultures as a function of time. Error bars represent standard deviation. Statistical significance was determined using an ordinary one-way ANOVA test followed by Dunnett’s multiple comparison test to compare each strain to the base strain

We compiled the genes of *K. phaffii* that are known components of the vacuolar pathway or have significant homology with vacuolar genes in *S. cerevisiae* (Table S10). Broadly, these genes include transporters, receptors, membrane proteins, and proteins involved in the formation, activation, or movement (including sorting) of other vacuolar proteins and vesicles. The essentiality profile for this group of vacuolar genes was very similar to that for the whole genome (Fig. 3A, Table S8).

We sorted the selected list by essentiality, and the 15 least essential genes were selected as knockout targets (Table S11). The essentialities of the selected vacuolar genes had a similar order of magnitude to those of the selected cell wall genes. We successfully disrupted all 15 genes in the base strain (S-63) producing HSA and the base strain (S-63) producing trastuzumab. The successful knockout strains were cultivated at plate scale for both proteins of interest. Seven of the 15 mAb-producing strains showed statistically significant improvements in specific productivity when compared to the base strain. An additional five of the 15 showed modest, though not significant, increases in productivity. The remaining three knockout strains had productivity similar to the base strain. The ΔGQ67_01477 and ΔVNX1 strains had comparable specific productivities to the ΔROT2, ΔATH1, ΔUTR2, ΔEXG1, and ΔYNL190W strains, showing 1.9-fold improvements over the base strain. ΔGQ67_05204 and ΔYPR109W were the highest-producing vacuolar strains with specific productivities comparable to ΔJSN1. They each showed a 2.4-fold improvement in specific productivity compared to the base strain (Fig. 3B). As observed with the strains with cell wall knockouts, the growth curves and profiles of host cell proteins for each vacuolar knockout strain were visually comparable (Fig. 3B, C).

All four of the genes in the highest-performing strains with vacuolar knockouts encode either vacuolar membrane proteins or membrane-localized proteins. Two of the four (YPR109W and GQ67_05204) are involved in vacuolar sorting. YPR109W is a sorting receptor for vacuolar hydrolases [27]. We hypothesize that disrupting this gene may directly suppress degradation pathways by reducing the availability of localized hydrolases. GQ67_05204 has an unknown function but is implicated in vacuolar protein sorting [27]. We hypothesize that disrupting this gene may reduce the number of proteins being directed through the vacuolar pathway, which, in turn, could reduce the degradation of desired products.

GQ67_01477 and VNX1 are membrane transporters. GQ67_01477 is an aminophospholipid translocase specifically associated with endocytosis and vacuolar biogenesis [27]. In *S. cerevisiae*, flippases have a reported role in protein transport [59]. We hypothesize that disruption of GQ67_01477 may directly affect trafficking of recombinant proteins by disrupting and redirecting vesicle pathways, thereby reducing internal sorting/degradation. Alternatively, this disruption could directly suppress pathways of degradation by limiting the transport of degradation-involved proteins. VNX1 encodes an antiporter (also known as a counter-transporter) for monovalent cations/H+ [27]. We hypothesize that disruption of this gene may influence the vacuolar and secretory pathways through altered pH or cation balance within the cell.

Unlike the strains with cell wall knockouts, some strains with vacuolar knockouts mildly modulated the production of HSA. ΔGQ67_01477, ΔGQ67_02083, and ΔTPM1 strains all produced a statistically significant effect on secretion (Figure S3). Interestingly, GQ67_01477 was the only gene disruption that caused a significant impact on the production of both trastuzumab and HSA. GQ67_02083 is a “phosphorylated vacuolar membrane protein that interacts with Atg13p” [27]. We hypothesize that disrupting this gene may impact the integrity of vacuolar membranes directly, thereby disrupting the vacuolar pathway. Alternatively, the disruption of this gene may affect the activity of Atg13p. In *S. cerevisiae*, Atg13p is a kinase required for vesicle formation and is specifically involved in vesicle formation for autophagy and the cytoplasm-to-vacuole targeting pathway [28]. We hypothesize that disruption of GQ67_02083 may indirectly impact vacuolar pathway trafficking by altering vesicle formation through changes to Atg13p activity. TPM1 is an isoform of tropomyosin that stabilizes actin cables and filaments [27]. We hypothesize that this protein could increase cell permeability or modify protein trafficking by altering the cell’s actin cytoskeleton.

Overall, the vacuolar edits modulated the production of HSA differently than they modulated the production of trastuzumab. Not only did the HSA expressing knockout strains experience less modulation overall, but the most impactful gene disruptions were not fully conserved across the two molecules. These results further support the hypothesis that HSA and trastuzumab generally experience different bottlenecks in production.

### Improvements translated to other cultivation methods

**Fig. 4:**
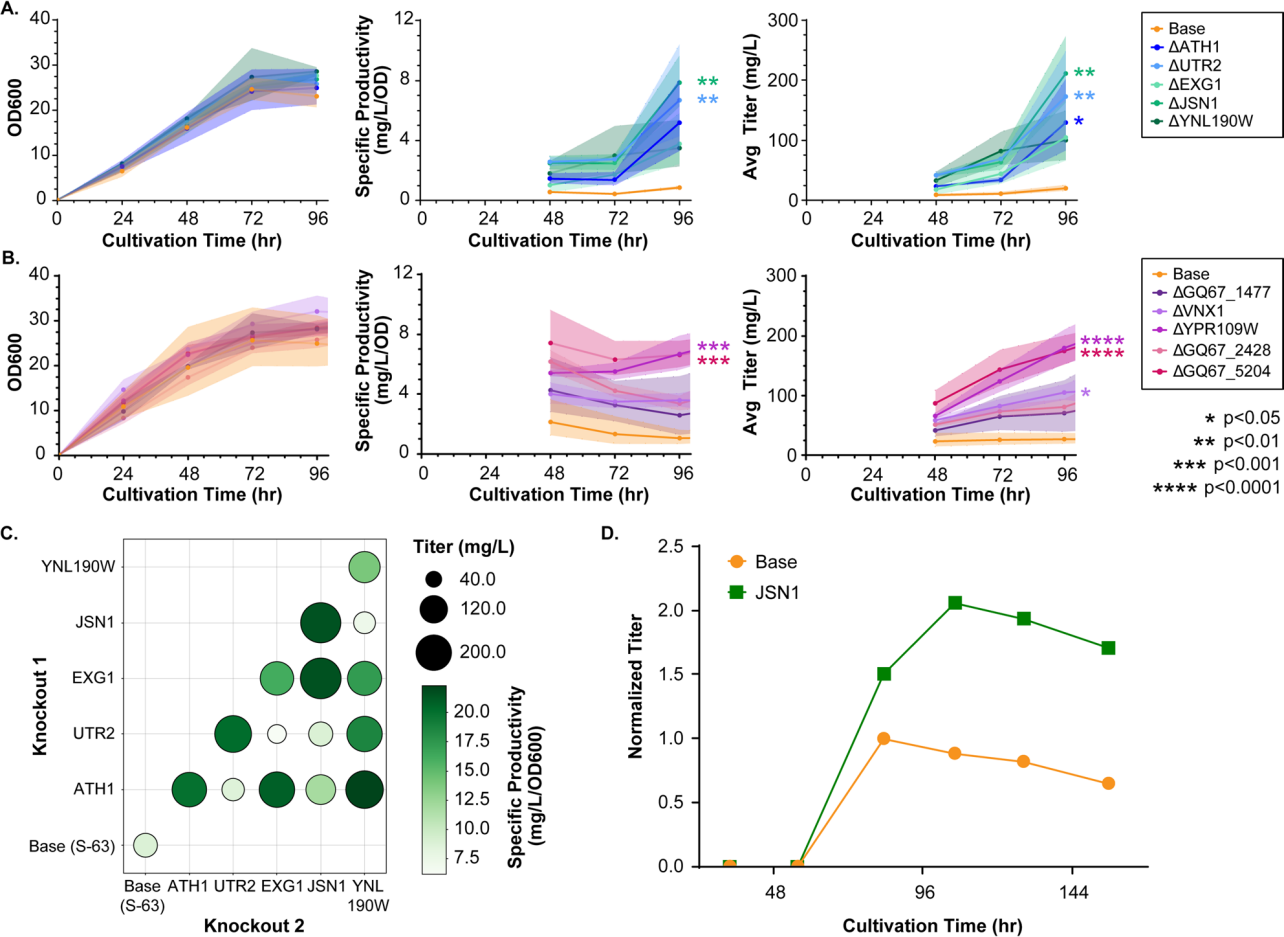
Characterization of best-performing strains in extended cultivations and in combination. **A** Optical densities (OD_600_), specific productivity (mg/L/OD_600_), and titers (mg/L) of strains with disrupted cell wall genes producing trastuzumab in extended 50 mL cultivation. Shaded error bars represent standard deviation. **B** Optical densities (OD_600_), specific productivity (mg/L/OD_600_), and titers (mg/L) of strains with disrupted vacuolar genes producing trastuzumab in extended 50 mL cultivation. Shaded error bars represent standard deviation. **C** Titers (mg/L) and specific productivities (mg/L/OD_600_) of strains with combined cell wall disruptions producing trastuzumab after 48 h of production in a 3 mL culture. **D** Normalized titers of trastuzumab produced by ΔJSN1 (cell wall disruption) and the base strain in a 250 mL fed-batch bioreactor (150 mL working volume), cultivated with defined media. Titers are normalized to that of the base strain titer at the first sampling time. Statistical significance was determined using an ordinary one-way ANOVA test followed by Dunnett’s multiple comparison test to compare each strain to the base strain (A and B) or the single knockout strain (C)

The performance of engineered strains often does not reliably translate across different methods and scales of cultivation [60]. Therefore, to validate the performance of the strains in other methods of cultivation and over an extended period, we selected the five trastuzumab-producing strains with the highest specific productivities from each gene group and cultivated those ten strains, along with the base strain, in batch in flasks. In the case of the strains with disrupted cell wall genes, we observed that the beneficial effects from the engineered strains were amplified during the cultivation (Fig. 4A). All strains maintained higher titers and specific productivities than the base strain throughout the cultivation, with statistically significant improvements observed for each engineered strain at one or more timepoints (Figure S4). ΔJSN1 and ΔUTR2 strains exhibited the highest titers and specific productivities with approximately 8–9 fold improvements measured at 96 h post-inoculation. There was no significant difference between the growth profiles or cellular mass of the selected strains (Fig. 4A). There was a small observed difference between the host cell protein profiles of the engineered strains and the base strain (Figure S4).

For the strains with modified vacuolar genes, the beneficial effects of the engineered strains were again maintained throughout cultivation in the flasks, with higher average titers and specific productivities observed for all strains (Fig. 4B, Figure S5). The engineered strains with vacuolar edits had slightly smaller gains than the strains with cell wall edits, with ΔYPR109W and ΔGQ67_05204 strains showing just over six-fold improvements in titers and specific productivities. The ΔGQ67_01477 strain was the only strain that did not exhibit statistically significant titers or specific productivities at any point during the cultivation. There were minimal differences between the host cell protein profiles of the strains (Figure S5). The profiles of host cell proteins from the flask cultures were visually similar for both the set of strains with cell wall disruptions and the set of strains with vacuolar disruptions (Figure S4, Figure S5). We also compared the titers achieved when cultivating the ΔJSN1 and base strains in fed-batch bioreactors with defined media. ΔJSN1 showed 2–3 fold improvements in titer (with comparable cell mass) when compared with the base strain (Fig. 4D).

### Strains with multiple gene disruptions did not show further productivity improvements

Individual gene disruptions from both pathways showed improved production of trastuzumab at multiple scales. We hypothesized that combining our best-performing knockout strains could further improve cell-specific productivity. We generated strains in which we disrupted all combinations of the five best cell wall-related genes and cultivated them for 72 h. Interestingly, the final titers and specific productivities of the combinations showed no significant improvements over single knockouts (Fig. 4C). In many cases, these combinations appeared to hinder the production of protein. Combinations of the disruptions for seven of the cell wall knockout strains and seven of the vacuolar knockout strains also showed similar results after a 48 h cultivation (Figure S6). All combinations had no observable differences in the profiles of growth and host cell proteins (Figure S7). These results suggest that further investigation is needed to understand how to combine disruptions to work synergistically. One hypothesis for the lack of compounding effects may be that the knockouts within these two gene groups all address a similar production bottleneck. The best-performing knockout strains across both gene groups (ΔJSN1, ΔGQ67_05204, and ΔYPR109W) all have disrupted genes that interact with the cellular membrane. If disrupting one of these genes alleviates a chokepoint in membrane permeability, augmenting membrane permeability through a second knockout may not increase productivity further. Once a specific bottleneck is addressed, a second genetic edit that targets a different rate-limiting step may be more effective.

## Discussion

The essentiality screen presented here is an efficient approach to estimate gene essentiality across a full genome. Comparisons with previously reported screens for essentiality, together with initial experimental validation, support the overall effectiveness of this method. This approach, however, may result in misclassification of genes. MCD4 may represent one example here since our attempts to disrupt it in a targeted manner were unsuccessful. Potential biological sources affecting the robustness of classification include reduced fitness upon the disruption of some non-essential genes, repairs to DNA that fail to generate loss-of-function mutations, and failure of the integrated Cas9 to make double-strand breaks due to inefficient gRNAs or limited accessibility of target loci [61, 62].

The arrayed CRISPR screen implemented in this work provided a structured format that facilitated easy and efficient screening of knockouts within multiple cellular pathways. Essentiality was effectively used as a metric to identify non-lethal candidates for disruption and to downselect individual genes from known pathways for further testing. All of the engineered strains displayed comparable profiles for growth and host cell proteins at multiple scales, indicating that disruptions of low-essentiality genes can have a minimal impact on viability. This method did not require detailed knowledge of the functions of the individual genes *a priori* and demonstrated how a quantifiable gene characteristic (essentiality) can facilitate increased engineering of less-annotated genes. In this study essentiality was only used to identify targets for gene disruption, though essentiality could also be used to inform other methods of genetic engineering, including gene up-regulation and insertion.

We successfully disrupted 29 non-essential genes associated with the cell wall and vacuolar transport. Of these single gene edits, 19 significantly improved the production of trastuzumab in *K. phaffii*. The strains with the largest observed increases in production (ΔJSN1, ΔGQ67_05204 and ΔYPR109W) yielded approximately 2.5-fold improvements at the 3 mL scale, and 6–9 fold improvements in flasks. The best performing strain, ΔJSN1, showed a three-fold improvement in production in a fed-batch reactor. Three knockouts (ΔGQ67_01477, ΔGQ67_02083 and ΔTPM1) significantly improved the production of HSA; only the ΔGQ67_01477 strain also improved the production of trastuzumab. The success of these engineered strains demonstrates the effectiveness of the methodology presented here for identifying new genetic engineering targets. This strategy should generalize to expand the catalog of beneficial engineering targets, and the strains created and characterized here can serve as base strains for subsequent layered engineering efforts.

The discrepancy between how the selected genetic edits affected the production of trastuzumab and the production of HSA suggests that different molecules have different bottlenecks in production, and that the knockouts explored in this study specifically benefit production of mAbs. Further targeting of genes from other biological pathways may highlight those that impact the production of HSA. Once key bottlenecks are identified and addressed for multiple molecules, it may be possible to engineer a common parental strain with several edits to alleviate common production bottlenecks and raise production across molecules.

We initially hypothesized that cell wall knockouts would improve cellular permeability, while vacuolar knockouts might reduce degradation of the desired product. The annotations of the top-performing knockouts appear to support these hypotheses; limited understanding of these genes, combined with the absence of targeted validation experiments, however, precludes definitive conclusions. Future studies using RNA sequencing, proteomics, or metabolic profiling may provide deeper insights into the biological mechanisms driving these improvements.

Of the gene edits tested, 86% of the cell wall-targeted disruptions, and 47% of the vacuolar-targeted ones, significantly increased the cell-specific productivity of trastuzumab. Further investigation is necessary to assess whether knockouts from selected gene groups result in a higher instance of titer improvements than randomly chosen non-essential gene disruptions. As the group of cell wall genes appeared to have a lower essentiality distribution than the group of vacuolar genes, it may be of interest to evaluate whether the essentiality distribution of a gene group is correlated with the percentage of beneficial knockouts.

For combination knockouts, further research is required to understand how to achieve synergistic effects when combining gene disruptions. The combinations tested here, together with the results of our secretome knockout study [26], indicate that rationally selected combinations of gene disruptions rarely exhibit linearly additive benefits. There are many combinations and pathways still unexplored. Additional studies to ascertain other relevant and quantifiable characteristics of genes (like expression level or essentiality with different carbon sources) may help identify synergistic interactions to inform engineering of combination knockouts when combined with the essentiality data reported.

## Conclusion

We have presented here a pooled CRISPR-Cas9 screen that predicted the probability of gene essentiality for every gene across the genome of *K. phaffii*. We disrupted low-essentiality genes from secretion-associated pathways (cell wall and vacuolar) and discovered 19 single gene edits that significantly improved the production of a recombinant mAb in *K. phaffii* with no discernible impacts on cell growth or secretory profile.

This study provides an engineering framework for combining data on gene essentiality and prior knowledge of biologic pathways related to a phenotypic trait of interest that can be expanded to any non-model organism. The published essentiality screen can provide an additional resource to enhance future genetic engineering efforts of *K. phaffii.* The modifications reported (and other modifications discovered using the methodologies developed here) could increase the volumetric productivity of non-model organisms, thereby facilitating the production of low-cost, high-quality recombinant proteins.

## Supplementary Information

Below is the link to the electronic supplementary material.

Supplementary Material 1.

Supplementary Material 2.

Supplementary Material 3.

Supplementary Material 4.

Supplementary Material 5.

Supplementary Material 6.

Supplementary Material 7.

Supplementary Material 8.

Supplementary Material 9.

Supplementary Material 10.

Supplementary Material 11.

Supplementary Material 12.

Supplementary Material 13.

Supplementary Material 14.

Supplementary Material 15.

Supplementary Material 16.

Supplementary Material 17.

## Data Availability

Plasmid sequences are included in the Supplemental Materials. Data from the analysis of the pooled DNA library is included in the Supplemental Materials.
