# Supplementary figures and images for: Deletion of low-essentiality, secretion-associated genes enhances recombinant protein production in Komagataella phaffii
Source: Microb Cell Fact. 2026 May 11;25:154. doi: 10.1186/s12934-026-03009-7 (PMC13343958; doi:10.1186/s12934-026-03009-7)

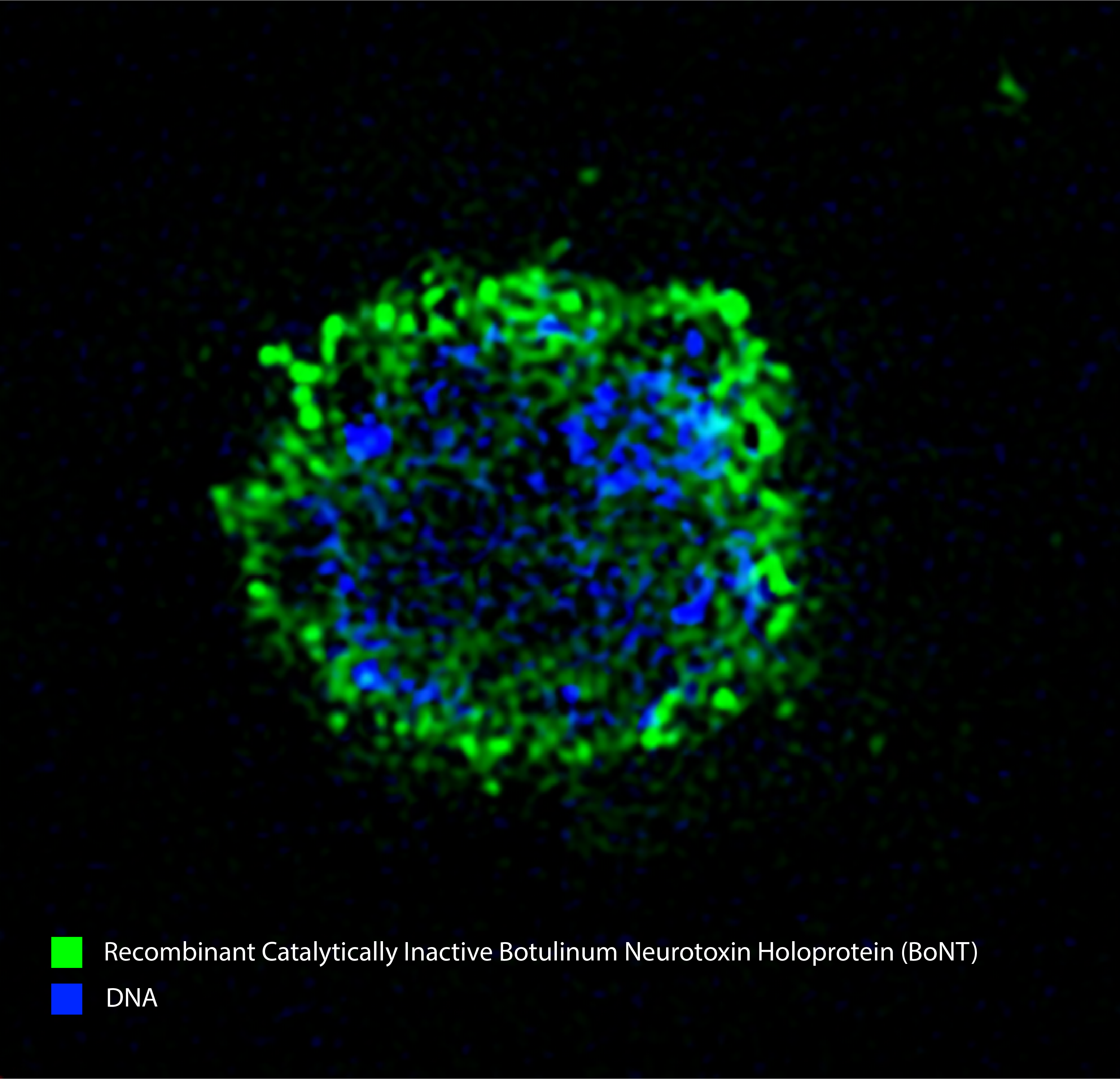

Supplement: Supplementary file 6 — Supplementary Material 6. [file 12934_2026_3009_MOESM6_ESM.png]
