# Supplementary material for: Deletion of low-essentiality, secretion-associated genes enhances recombinant protein production in Komagataella phaffii
Source: Microb Cell Fact. 2026 May 11;25:154. doi: 10.1186/s12934-026-03009-7 (PMC13343958; doi:10.1186/s12934-026-03009-7)
Supplement: Supplementary file 17 — Supplementary Material 17. [file 12934_2026_3009_MOESM17_ESM.docx]

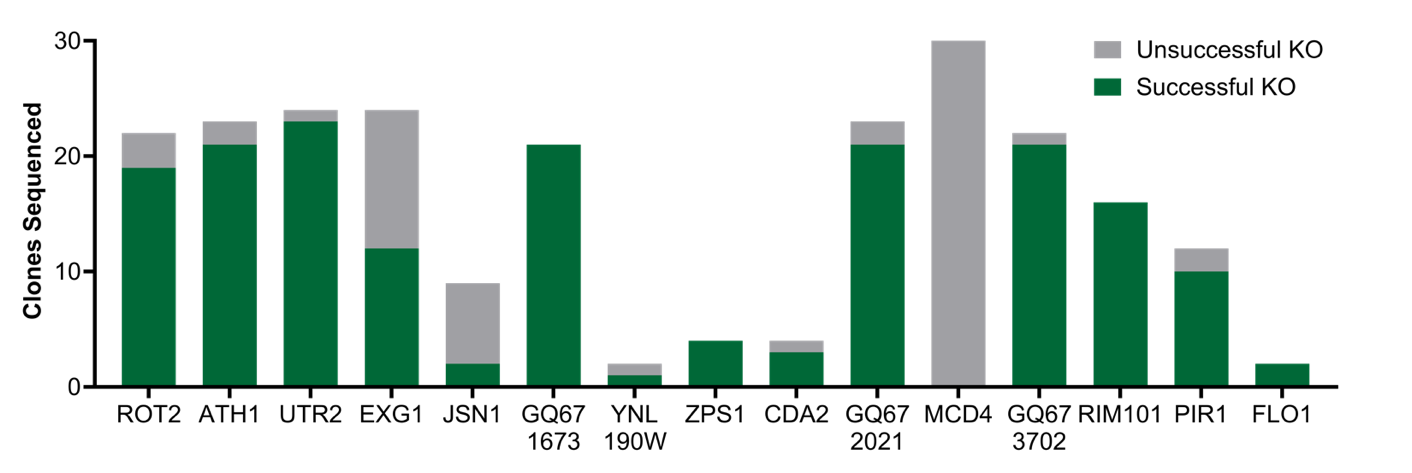


**Figure S1** Success rates of cell wall gene disruptions. A successful disruption was defined as any disruption with a genetic edit at the CRISPR-Cas9 cut site that caused a premature stop codon. Unsuccessful disruptions included strains with no genetic edits and strains with genetic edits that did not cause premature stop codons.

**
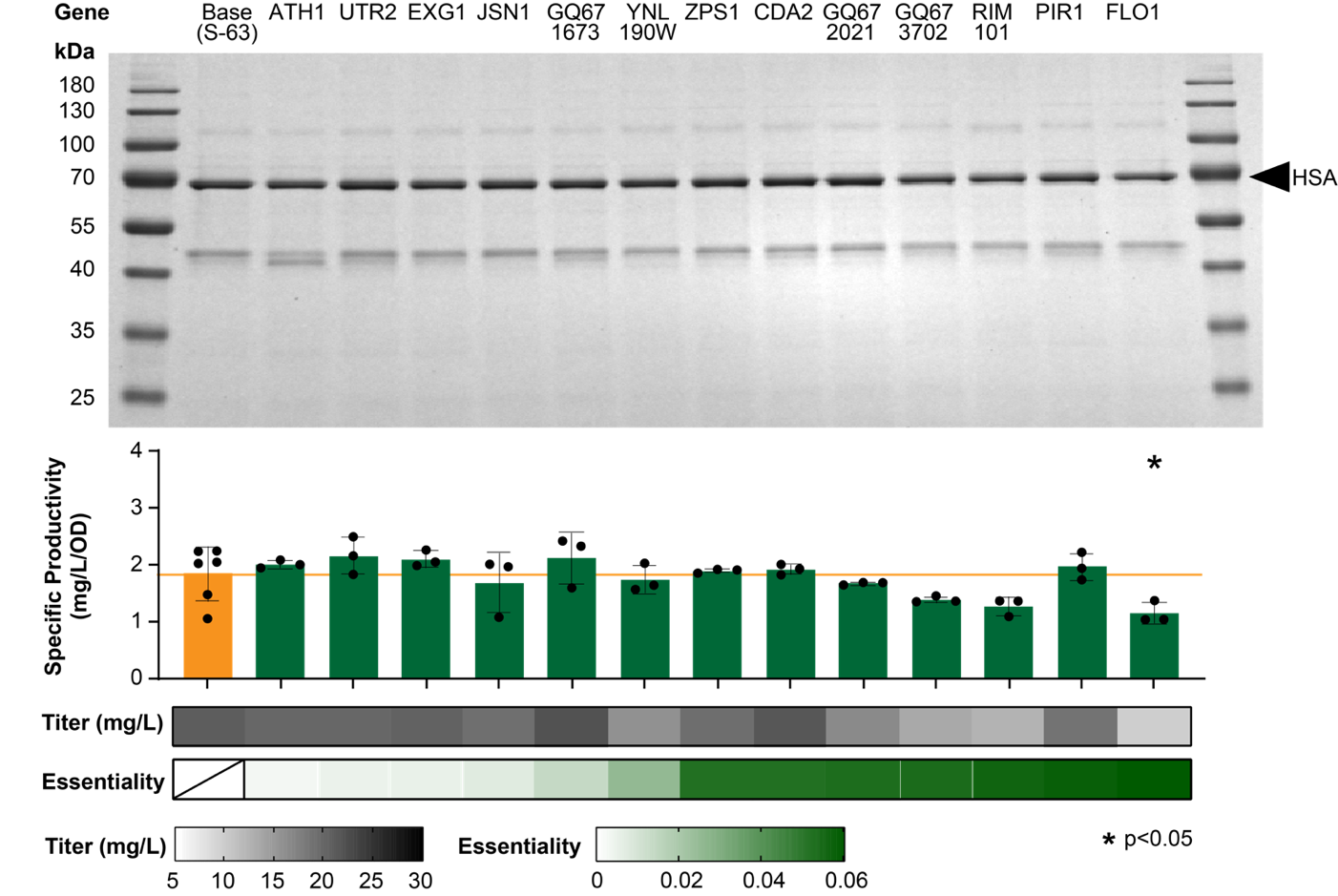
**

**Figure S2** Effects of targeted cell wall gene disruptions on the secretion of HSA. Results include SDS-PAGE gel of supernatants from 3 mL cultures, corresponding specific productivities (mg/L/OD_600_), titers (mg/L), and gene knockout essentiality scores. Error bars represent standard deviation. Statistical significance was determined using an ordinary one-way ANOVA test followed by Dunnett’s multiple comparison test to compare each strain to the base strain.


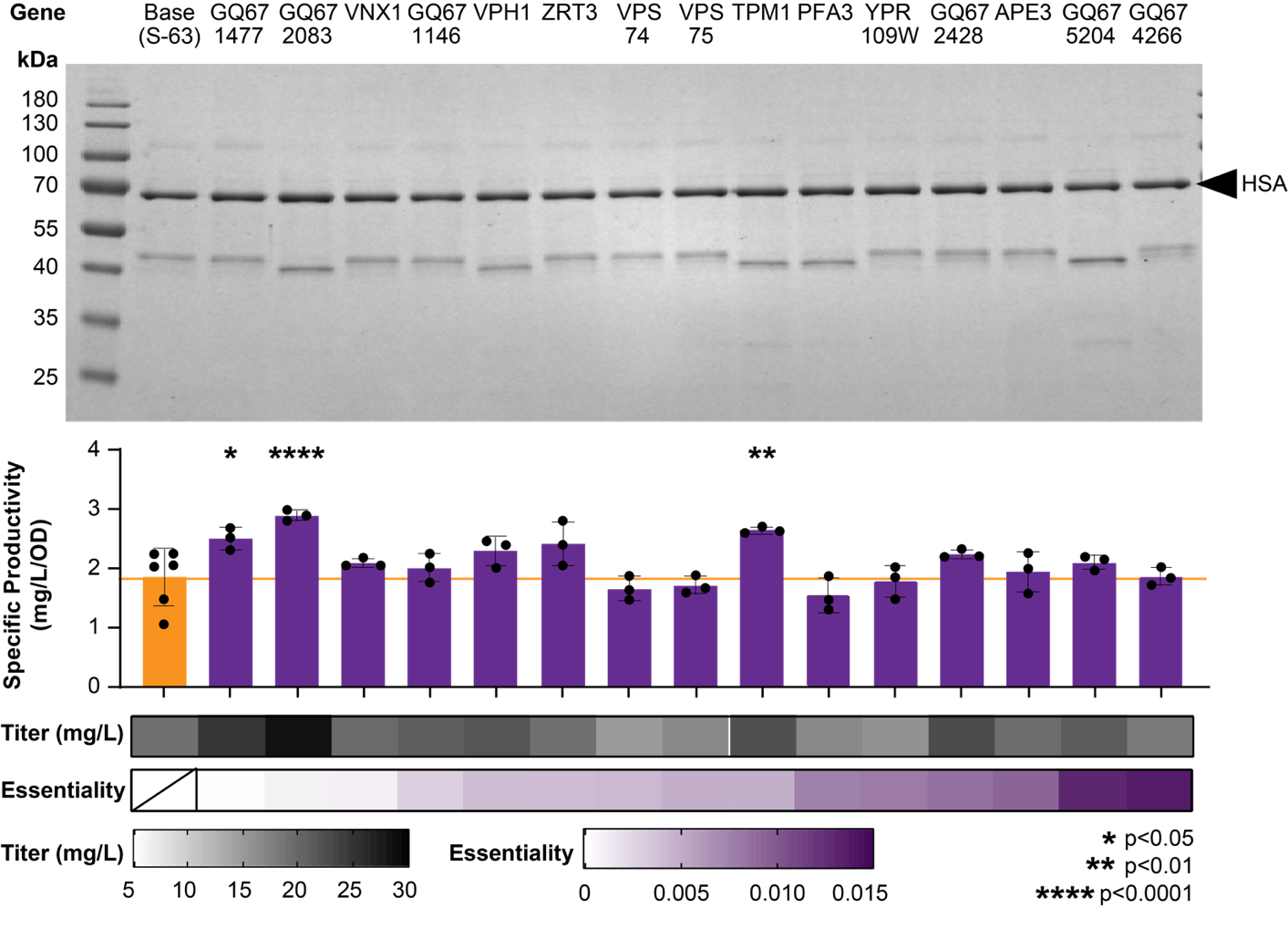


**Figure S3** Effects of targeted vacuolar gene disruptions on the secretion of HSA. Results include SDS-PAGE gel of supernatants from 3 mL cultures, corresponding specific productivities (mg/L/OD_600_), titers (mg/L), and gene knockout essentiality scores. Error bars represent standard deviation. Statistical significance was determined using an ordinary one-way ANOVA test followed by Dunnett’s multiple comparison test to compare each strain to the base strain.

**
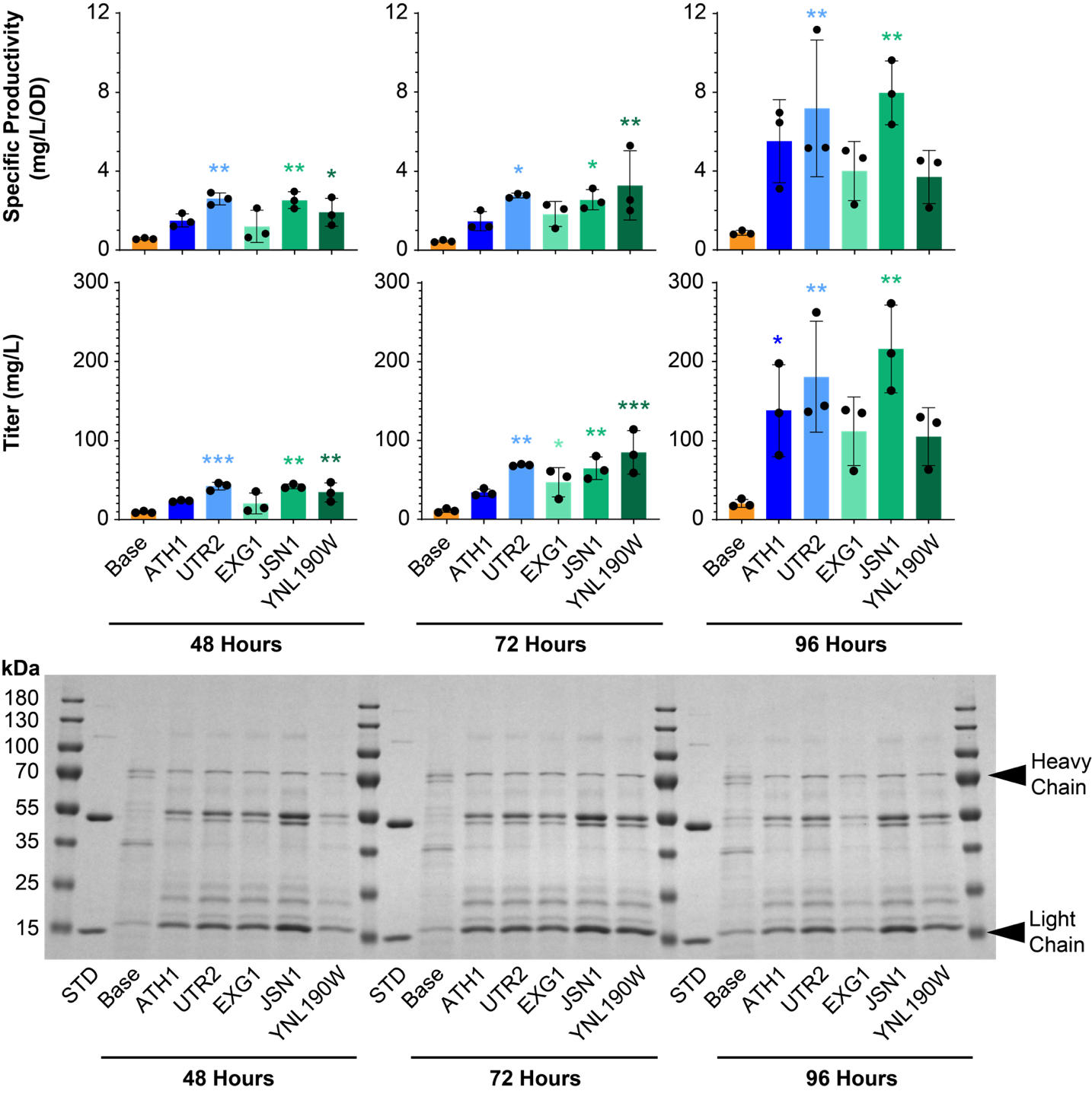
**

**Figure S4** Characterization of extended 50 mL flask cultivation for best cell wall gene disruption strains. Data shown includes specific productivity (mg/L/OD_600_), titer (mg/L) and SDS-PAGE gels for each gene disruption strain at various points throughout the cultivation. Error bars denote specific productivity. * Denotes significance at p ≤ 0.05. ** Denotes significance at p ≤ 0.01. ***Denotes significance at p ≤ 0.001. Statistical significance was determined using an ordinary one-way ANOVA test followed by Dunnett’s multiple comparison test to compare each strain to the base strain.

**
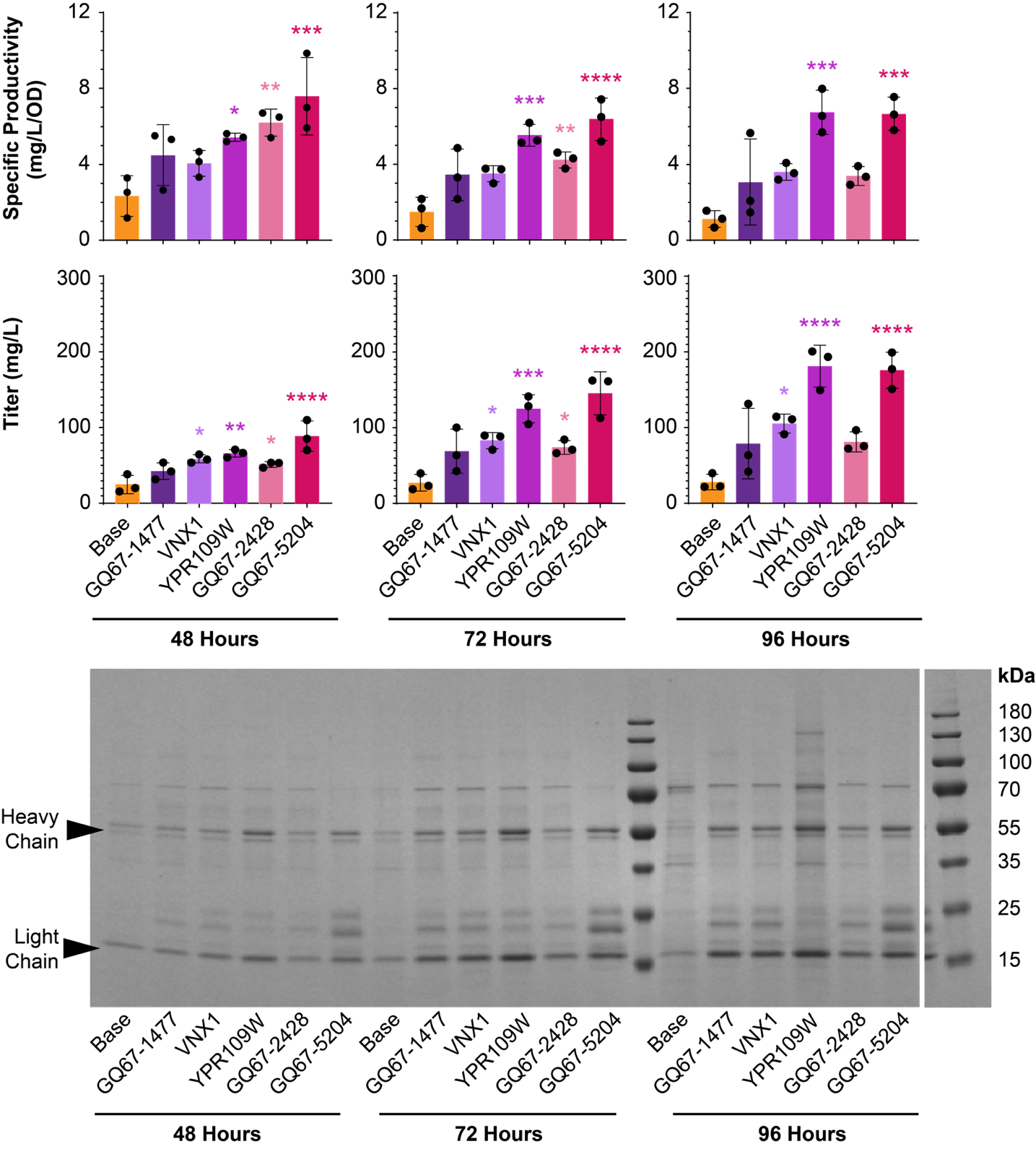
**

**Figure S5** Characterization of extended 50 mL flask cultivation for best vacuolar gene disruption strains. Data shown includes specific productivity (mg/L/OD_600_), titer (mg/L) and SDS-PAGE gels for each gene disruption strain at various points throughout the cultivation. Error bars denote specific productivity. * Denotes significance at p ≤ 0.05. ** Denotes significance at p ≤ 0.01. ***Denotes significance at p ≤ 0.001. Statistical significance was determined using an ordinary one-way ANOVA test followed by Dunnett’s multiple comparison test to compare each strain to the base strain.


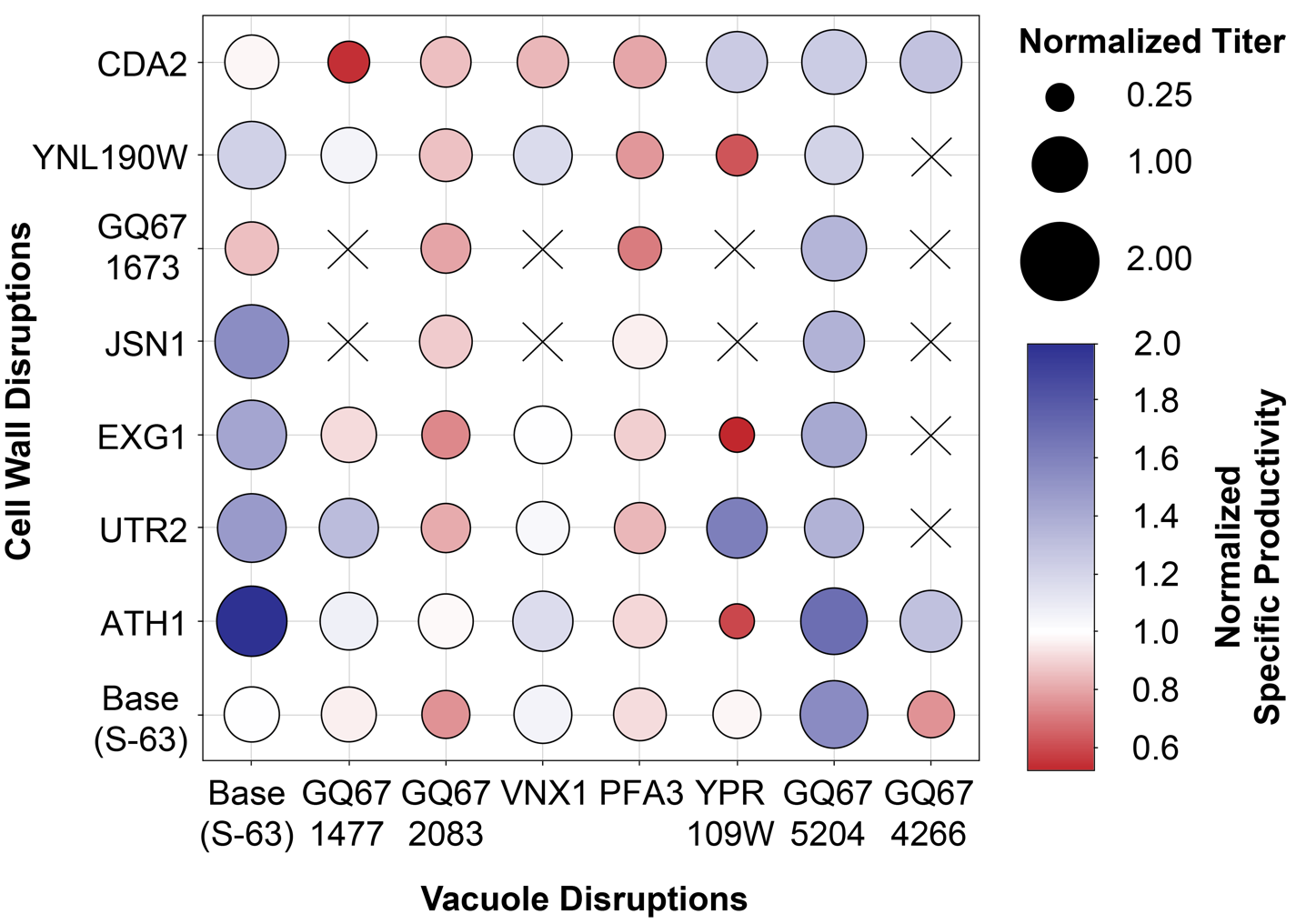


**Figure S6** Characterization of cell wall and vacuolar combination gene disruptions. Missing combination disruptions (represented as X) had unsuccessful integration of the second disruption. Reported titers and specific productivities have been normalized with respect to the base strain. Statistical significance was determined using a two-way ANOVA test followed by Dunnett’s multiple comparison test comparing data from each combination knockout to both of its parent strains.

**
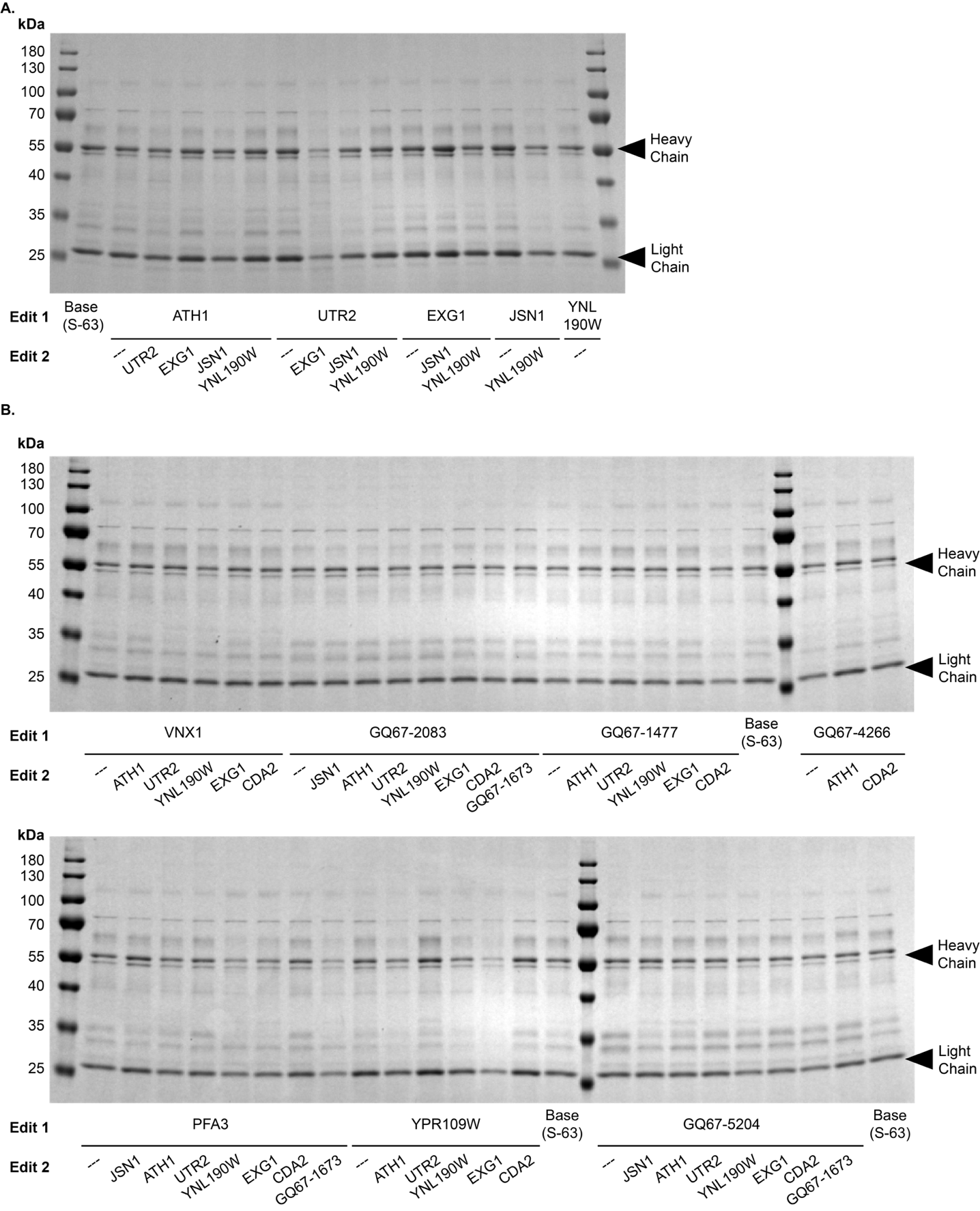
**

**Figure S7** SDS-PAGE gels of combination gene disruptions. **A.** SDS-PAGE gel of cell wall combination disruptions after 48 hours of production. **B.** SDS-PAGE gel of cell wall and vacuolar combination disruptions after 24 hours of production.
